# *Arabidopsis* mutants in sphingolipid synthesis as tools to understand the structure and function of membrane microdomains in plasmodesmata

**DOI:** 10.3389/fpls.2014.00003

**Published:** 2014-01-24

**Authors:** Ariadna González-Solís, Dora L. Cano-Ramírez, Francisco Morales-Cedillo, Cinthya Tapia de Aquino, Marina Gavilanes-Ruiz

**Affiliations:** Departamento de Bioquímica, Facultad de Química, Universidad Nacional Autónoma de MexicoMexico City, Mexico

**Keywords:** sphingolipid *Arabidopsis* mutants, sphingolipids and microdomains, long chain bases, sphingoid bases, microdomains and plasmodesmata

## Abstract

Plasmodesmata—intercellular channels that communicate adjacent cells—possess complex membranous structures. Recent evidences indicate that plasmodesmata contain membrane microdomains. In order to understand how these submembrane regions collaborate to plasmodesmata function, it is necessary to characterize their size, composition and dynamics. An approach that can shed light on these microdomain features is based on the use of *Arabidopsis* mutants in sphingolipid synthesis. Sphingolipids are canonical components of microdomains together with sterols and some glycerolipids. Moreover, sphingolipids are transducers in pathways that display programmed cell death as a defense mechanism against pathogens. The study of *Arabidopsis* mutants would allow determining which structural features of the sphingolipids are important for the formation and stability of microdomains, and if defense signaling networks using sphingoid bases as second messengers are associated to plasmodesmata operation. Such studies need to be complemented by analysis of the ultrastructure and the use of protein probes for plasmodesmata microdomains and may constitute a very valuable source of information to analyze these membrane structures.

## Introduction

Plasmodesmata (PD) are specialized membranous structures that allow the communication among contiguous plant cells, originating interconnected symplastic domains. Communication arise through these intercellular pores that allow the exchange of small molecules, such as ions, sugars, phytohormones and macromolecules -RNA, transcription factors, even virus (Kim and Zambrisky, 2005) and effectors derived from pathogens (Lewis et al., 2009). This selective intercellular flow of molecules follows a defined direction and occurs at precise developmental stages or during stress responses (Kragler, 2013).

**Figure 1:**
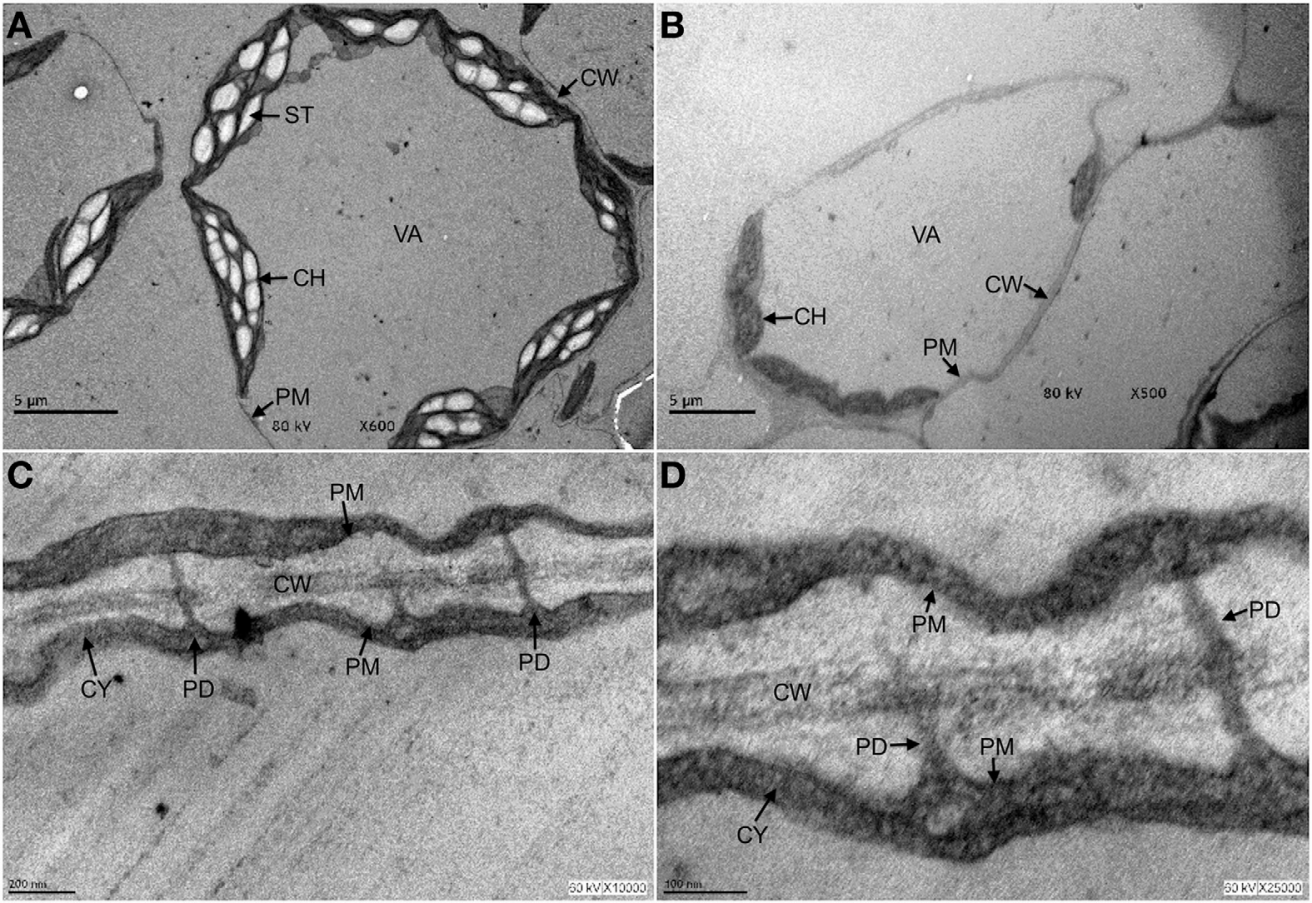
**Plasmodesmata are structures that persist under autophagy conditions**. Three-week old *Arabidopsis* seedlings were exposed to 10 μ M fumonisin B1 for 4 days in order to induce programmed cell death in the form of autophagy. After this time, leaf tissue was fixed and processed for transmission electron microscopy analysis as described in Saucedo-García et al. (2011a,b). **(A)** Control leaves from seedlings exposed to H2O. **(B–D)** Leaves from seedlings exposed to fumonisin B1 are shown at the indicated magnification. In **(A)**, it is observed that under control treatment, cells show a rounded shape with typically elongated chloroplasts and starch bodies, some other small organelles in the periphery and well defined plasma, vacuole and chloroplasts membranes. In **(B)**, cells from seedlings exposed to fumonisin B1 show undergoing autophagy at different stages: some cells are already empty and only the cell walls reveal their former presence; a remaining cell still displays no visible organelles, chloroplasts but with smaller size and undefined membranes. In **(C,D)**, magnifications of the FB1-treated seedlings show that cells undergoing autophagy and with few cell remnants still clearly exhibit cell walls and PD structures. CH, chloroplast; CW, cell wall; CY, cytosol; PD, plasmodesmata; PM, plasma membrane; ST, starch; VA, vacuole.

Imaging techniques that allow preservation of PD structure revealed a very complex and refined organization, but its molecular composition is difficult to dissect by biochemical approaches (Brunkard et al., 2013; Salmon and Bayer, 2013). However, PD are stable assemblies that can be found in cell wall preparations (Brecknock et al., 2011; Salmon and Bayer, 2013) and can even survive treatments involving cell autophagy (Figure 1). PD are formed by the extension of the PM of two adjacent cells, containing a central cylinder constituted by the prolongation of the endoplasmic reticulum (ER) of the joint cells. This ER is embedded in a cytoplasmic milieu common to the interconnected cells. Insoluble glycans as callose are deposited in the neck of the structure (Maule et al., 2011).

While many of the proteins present in the PD are known (Fernandez-Calvino et al., 2011; Raffaele et al., 2009), few studies have dealt with the lipid phase from PD (Cacas et al., 2012). Recent evidences suggest the presence of membrane microdomains in the PM of the PD (Tilsner et al., 2013). Remorin, a key protein identified and considered a marker of plant PM microdomains is present in PD (Raffaele et al., 2009; Mongrand et al., 2010). Moreover, glycosylphosphatidylinositol (GPI) anchored proteins frequently found in PM microdomains (Brown and Rose, 1992; Schroeder et al., 1994) have been localized in the PD through subcellular fractionation and proteomic analysis (Fernandez-Calvino et al., 2011; Simpson et al., 2009; Salmon and Bayer, 2013). In addition, the presence of phytosterols, canonical lipid components of microdomains (Mongrand et al., 2004; Laloi et al., 2007) was inferred from experiments in which treatment with a sequestering sterol compound promotes the relocalization of remorin from the detergent insoluble membranes to the detergent soluble membrane fraction and a change of its distribution from clusters (microdomain organization) to random positions (Raffaele et al., 2009). These evidences suggest the presence of microdomains in the PD and imply, but do not assure, that the general structural design of PD microdomains follows the same principles operating in known membrane domains.

In plants, as in other eukaryotes, sphingolipids, together with sterols are essential constituents of membrane microdomains (Mongrand et al., 2004; Sperling et al., 2005; Laloi et al., 2007; Carmona-Salazar et al., 2011; Cacas et al., 2012). Their chemical structure, with a highly hydrophilic polar head and a hydrophobic moiety formed by a long chain fatty acid and a sphingoid base or long chain base (LCB), makes them ideal candidates to form tightly packed regions in the membrane. These highly-ordered phases segregate in the bulk of the more extended fluid region of the membrane. Moreover, sphingolipids are very diverse due to the chemical variants of the three moieties; lipidomic analyses from *Arabidopsis thaliana* revealed about 300 molecular species of these complex lipids (Markham et al., 2006; Markham and Jaworski, 2007) Such diversity is generated by the biosynthetic enzymes and others catalyzing chemical modifications such as hydroxylation, desaturation and phosphorylation (Markham et al., 2013). T-DNA insertion mutants and gene silencing of genes coding for these enzymes have contributed to reveal the role of different sphingolipid species (Table 1). Plant cells require this enormous diversity in sphingolipids to carry out specific cell tasks that involve structural and signaling aspects (Chen et al., 2006; Dietrich et al., 2008; Chao et al., 2011; Saucedo-García et al., 2011a; König et al., 2012). For instance, the LCB dihydrosphingosine and a hydroxylated ceramide are involved in the programmed cell death as part of the immune response (Liang et al., 2003; Shi et al., 2007; Wang et al., 2008), the LCB sphingosine 1-P is a mediator in stomata closure in *Commelina communis* (Ng et al., 2001), but is phytosphingosine 1-P which displays this role in *Arabidopsis* (Coursol et al., 2003). This phosphorylated LCB is also produced in response to low temperatures (Cantrel et al., 2011; Dutilleul et al., 2012), while complex sphingolipids built from desaturated LCB lead to aluminum and cold tolerance in *Arabidopsis* (Ryan et al., 2007; Chen et al., 2012). A great variety of complex sphingolipids is involved in the formation of the lipid bilayer and its microdomains in the plant membranes (Sperling et al., 2005; Mongrand et al., 2010).

**Table 1:** ***Arabidopsis* mutants impaired in sphingolipid metabolism**.

**Mutant**	**Modified gene, encoded protein**	**Gene ID**	**Characteristics**	**References**
*fbr11-1 ( lcb1)*	*LCB1*, subunit of serine palmitoyltransferase (SPT)	At4g36480	Reduced sensitivity to FB1-induced cell death	Chen et al., 2006; Shi et al., 2007
*lcb2a*	*LCB2a*, subunit of serine palmitoyltransferase (SPT)	At5g23670	Implicated in male gametogenesis and embryogenesis. Reduced sensitivity to FB1.	Dietrich et al., 2008; Saucedo-García et al., 2011a,b
*Atlcb2b hp/Atlcb2a*	*LCB2a LCB2b*, subunits of serine palmitoyltransferase (SPT)	At5g23670/ At3g48780	Inducible silencing of *Atlcb2b* in a *Atlcb2a* mutant background. Reduced total sphingolipid content upon induction with methoxyfenozide	Dietrich et al., 2008
*tsc10a*	*TSC10A*, 3-ketodihydrosphinganine reductase	At3g06060	Increased content of Na, K and Rb ions and decreased levels of Mg, Ca, Fe and Mo. Contributes with > 95% of the 3-KDS reductase activity in *Arabidopsis*.	Chao et al., 2011
*tsc10b*	*TSC10B*, 3-ketodihydrosphinganine reductase	At5g19200	Decreased content of K and Rb ions and increased Ca and Mo	Chao et al., 2011
*loh1*	*LOH1*, very-long-acyl-chain ceramide synthase (CS II)	At3g25540	Complete depletion of ceramides with a fatty acid acyl chain longer than C18 and excessive amounts of sphingolipids containing C16:0	Markham et al., 2011
*loh2*	*LOH2*, long-acyl-chain ceramide synthase (CS I)	At3g19260	Depletion of sphingolipids with fatty acids of 16 C. High sensitivity to FB1 and AAL toxin	Markham et al., 2011
*loh3*	*LOH3*, very-long-acyl-chain ceramide synthase (CS II)	At1g13580	Complete depletion of ceramides with fatty acid acyl chains longer than C18 and excessive amounts of sphingolipids containing 16:0	Markham et al., 2011
*sld1*	*SLD1*, Δ8 desaturase	At3g61580	Large reduction of Δ8 unsaturated LCB Reduction in glucosylceramide levels and increase in glycosyl inositolphosphoceramides	Chen et al., 2012
*sld2*	*SLD2*, Δ8 desaturase	At2g46210	Little reduction of Δ8 unsaturated LC. Reduction in glucosylceramide levels and increase in glycosyl inositolphosphoceramides	Chen et al., 2012
*sld1-sld2*	*SLD1*, Δ8 desaturase- SLD2 Δ8 desaturase	At3g61580/ At2g46210	Enhanced sensitivity to low temperature, grown at 0°C shows premature senescense and chlorotic lesions. Reduction in glucosylceramide levels and increase in glycosyl insitolphosphoceramides	Chen et al., 2012
Δ*4 des*	Δ*4 DES*, Δ4 desaturase of LCB	At4g04930	Selective expression in flower and pollen. Reduced content of glucosylceramide in flowers. Channeling of substrates to the glucosylceramide synthesis	Michaelson et al., 2009
*ads2*	*ADS2*, acyl-CoA desaturase	At2g31360	Reduced levels of 24:1-CoA and 26:1-CoA	Smith et al., 2013
*sbh1*	*SBH1*, C4-hydroxylase of LCB	At1g69640	Reduced content of trihydroxy LCB	Chen et al., 2008
*sbh2*	*SBH2*, C4-hydroxylase of LCB	At1g14290	Reduced content of trihydroxy LCB	Chen et al., 2008
*sbh1/sbh2*	*SBH1*, C4-hydroxylase- SBH2, C4-hydroxylase of LCB	At1g69640/ At1g14290	Lack of trihydroxy LCB. Accumulation of total sphingolipids with predominantly C16 fatty acids. Spontaneous programmed cell death. Defects in cell elongation and cell division	Chen et al., 2008
*fah1*	*FAH1*, hydroxylase of FA	At2g34770	Reduced content of sphigolipids with Δ-hydroxylated fatty acids	König et al., 2012
*fah2*	*FAH2*, hydroxylase of FA	At4g20870	Reduced content of sphingolipids with Δ-hydroxylated fatty acids	König et al., 2012
*fah1/fah2*	*FAH1*, hydroxylase- FAH2, hydroxylase of FA	At2g34770/ At4g20870	Increased ceramide and salycilate levels. Reduced leaf and root growth.	König et al., 2012
			Enhanced resistance to biotrophic pathogens	
*erh1*	*ERH1*, inositolphosphorylceramide synthase (IPCS)	At2g37940	Enhanced transcription of *RPW8* and RPW8-dependent spontaneous HR-like cell death in leaf tissues, and reduction in plant height. Salicylic acid accumulation	Wang et al., 2008
*lcbk2*	*LCBK2*, LCB kinase	At2g46090	Involved in the phosphorylation of LCB in chilling response	Dutilleul et al., 2012
*sphk1*	SPHK, sphingosine kinase	At4g21540	Involved in guard cell ABA signaling and seed germination	Coursol et al., 2003; Worrall et al., 2008
*acd5*	CERK, ceramide kinase	At5g51290	Increased content of ceramide, susceptibility to pathogen infection	Liang et al., 2003

### *Arabidopsis* mutants, complex sphingolipids and the PD microdomain structure

Due to the instability (half-life 10–20 ms, Eggeling et al., 2009) and size (about 100 nm, Raffaele et al., 2009; Demir et al., 2013) microdomains are membrane zones difficult to study. Moreover, analysis of microdomains in complex structures such as the PD imposes additional problems to determine their size, distribution, and function Cacas et al., 2012. Recently developed techniques based on molecular interactions and tracking of fluorescent particles may give more information of the PD microdomains (Jacobson et al., 2007; Pike, 2009; Lingwood and Simons, 2010). Given the high elusiveness of the structure and organization of PD, microdomain studies require other approaches as the use of *Arabidopsis* mutants in genes coding enzymes of sphingolipid synthesis. This can be a very helpful strategy to dissect structural and functional features of PD microdomains and PD membranes as well.

### Significance of sphingolipids in PD microdomains

It is expected that PD microdomains are mainly composed of sphingolipids and sterols as other plant PM microdomains but the factual contribution of sphingolipids to the PD structure and function is unknown. Mutants with a significantly reduced content of total sphingolipids may show low abundance or reduced size of PD and PD microdomains; this can be visualized using remorin and imaging measurements with high resolution microscopy. Some characterized *Arabidopsis* mutants that are ideal for this purpose, such as line *Atlcb2b hp/Atlcb2a*, a silenceable mutant in the serine palmitoyltransferase or SPT (first enzyme of the sphingolipid synthesis), containing 64% of total sphingolipids (Dietrich et al., 2008). Other mutant lines, *tsc10a and tsc10b*, in the keto-sphinganine reductase gene (second enzyme in the sphingolipid synthesis) contain only 10% of these lipids (Chao et al., 2011). The wide difference in the total sphingolipid content between these mutants provides an opportunity to estimate the quantitative involvement of these complex lipids on the structure of the PD and PD microdomains.

### Importance of the hydroxylated groups from the acyl chains

The high cohesion degree of sphingolipids and sterols that contributes to the formation of the Lo (liquid-ordered phase) characteristic of the membrane microdomains is mainly due to the harmonizing sterical shapes of sphingolipids and sterols, and the cooperative hydrophobic forces between them, and also to the hydrogen bonding at their polar regions, specially those close to the hydrophobic tails. In this zone, the presence of charged and polar groups from the LCB, fatty acids and sterols, provide the hydrogen bonding between sterols and sphingolipids that contributes to strengthen a membrane domain. Mutants impaired in the hydroxylation of sphingolipids, such as the sphingoid base hydroxylases SBH1 and SBH2 (*sbh1-1* and *sbh2-1* mutants, respectively) (Chen et al., 2008) or fatty acid hydroxylases FAH1 and FAH2 (*fah1-fah2* mutants, respectively) (König et al., 2012) can be helpful in this matter.

### Importance of the double bonds from the acyl chains

Saturated and all-trans acyl chains from fatty acids and LCB from sphingolipids favor their interaction with the planar sterol ring system producing a tight packing effect that characterizes the Lo phase of microdomains (Simons and Vaz, 2004). Mutants with different expression of desaturases of the sphingoid chain, such as lines Δ*4 des, sld1* and *sld2* (Sperling et al., 1998; Michaelson et al., 2009; Chen et al., 2012) or mutants in the fatty acid desaturase of sphingolipids, such as line *ads2* (Smith et al., 2013) may shed light on the relevance of the presence of saturated acyl chains to the configuration and stability of the PD microdomains.

### Relevance of the sphingolipid polar head

Caveolae, bottle-like membrane structures are a well-characterized case of stable membrane domain. They show a neck which formation is favored by the asymmetric and dense presence of sphingolipids in the outer monolayer of the membrane. This lipid effect has been explained by the carbohydrate voluminous hydrophilic heads and by the tight packing of their acyl chain region with cholesterol, promoting and stabilizing the bending of the membrane to originate the curvature of the caveolae entrance (Dart, 2010). It is possible that the asymmetric distribution of sphingolipids recruited at the inner PM monolayer at both sites of the PD entrance could accentuate the curvature of the PM. Callose deposition at these points seems to contribute to this deformation (Maule et al., 2011) that regulates the aperture of the PD (Roberts and Oparka, 2003; Epel, 2009). Another protein involved in the callose deposition is Plasmodesmata-Callose-Binding-Protein 1 (PDCB1), which could function as a structural anchor between the cell wall and the PM components of PD (Simpson et al., 2009). Taking into account that protein-protein interactions constitute a stabilization force in microdomains, PDCB1 could participate in the recruitment of lipids forming microdomains at or near the neck (Tilsner et al., 2013). The use of sphingolipid mutants would be useful to elucidate the interactions among lipid-protein-callose. In addition, the mutant *erh1*, which contains an imbalanced content of inositolphosphoceramide species (Wang et al., 2008) or the mutant *sld1* and *sld2*, which has low amount of glucosylceramide but an increased amount of glycosyl inositolphosphoceramides (Chen et al., 2012) may help to reveal the features of the sphingolipid polar heads that are significant to form the PD and their microdomains. Thus, residence of specific proteins and lipids in these membrane regions could favor the convexity of the inner monolayer of the PM (Voeltz and Prinz, 2007; Shibata et al., 2009).

### Relevance of sphingolipids in the recruitment of proteins to membrane microdomains involved in defense responses

It has recently been shown that key elements in regulating the flux through the PD pore are proteins like Lysin Motif Domain-Containing Glycosylphosphatidylinositol-Anchored Protein 2 (LYM2) and Plasmodesmata-Located-Protein 5 (PDLP5) which are enriched in PD membranes. LYM2 mediates the reduction of the aperture of the PD pore in the presence of the Pathogen-Associated-Molecular-Pattern (PAMP) chitin (Faulkner et al., 2013) and PDLP5 controls the permeability during bacterial infections. In the case of PDLP5, the accumulation of the phytohormone salicylic acid elicits the over-expression of this PD protein and increases the deposition of the 1,3-glucan polymer callose, reducing the PD orifice dimension (Lee et al., 2011; Wang et al., 2013). The fact that the PDLP5 resides exclusively in the center of the PD cavity, reinforces the hypothesis that this protein is located in microdomains. Signaling mechanism controlling PD function has been suggested (Brunkard et al., 2013) but it is possible that the lipidic environment is essential for certain proteins in order to keep its position in PD. In this regard, the hypothesis is that the components of the PM could sense the accumulation of the protein in order to initiate the response that will recruit callose synthases (Wang et al., 2013). One approach to investigate the role of microdomains in determining the function of the PDLP5 would be working with mutants defective in genes linked to sphingolipid metabolism and exploring whether disruption in the lipid environment of this protein affect the closure of the pore. In this respect, the use of *Arabidopsis* mutants as *Atlcb2b hp/Atlcb2a* and *tsc10a* which contain less complex sphingolipids could help to elucidate the role of sphingolipids as regulatory structures that affect PD membrane proteins.

### *Arabidopsis* mutants, LCB and the PD function in defense responses against pathogens

One of the main features of the signal transduction pathways is the fast intracellular transmission of the message triggered by the initial stimulus. In many cases, this propagation can proceed beyond, moving forward to some neighbor cells or reaching even long distances to become systemic information. PD, as universal connecting pores in plant tissues, mediate the diffusion of toxic and signaling molecules. However, this flux is selective and regulated. The size exclusion limit of PD is controlled during the developmental stage of the plant and upon pathogen infection (Angell et al., 1996; Xu et al., 2012). This is necessary to limit the flux of pathogenic molecules that might disturb neighboring cells and therefore determine disease susceptibility. However, at the same time, the intercellular communication regarding other defense molecules such as sRNAs must persist in order to establish a systemic response of resistance. In this direction, it has been demonstrated that the synthesis of siRNAs (silencing RNAs) as a response to a viral infection is a very effective systemic defense reaction in plants (Marín-González and Suárez-López, 2012; Parent et al., 2012). In addition, it has been shown that an increase in callose deposition affects the signaling mediated by miRNAs (Vatén et al., 2011) and that miRNAs are involved in defense against bacterial PAMP as well (Parent et al., 2012). It is reasonable to expect that the control of the flow under infection conditions depends on intrinsic characteristics of the PD pore determining its size and selectivity, but also from other extrinsic ones, as concentration gradients of transit molecules as sRNAs. In fact, some of these regulatory factors originate in the chloroplast (Brunkard et al., 2013). Regarding the residence of molecules responsible of the signaling implied in the regulation of the aperture/closure of the PD pore, a significant number of receptor-like kinases has been described in PD proteomic studies (Fernandez-Calvino et al., 2011), thus suggesting that these proteins may have a crucial role in determining the changes of PD structure upon infection (Lee and Lu, 2011). In this context, it is appealing the idea of exploring the structural role of sphingolipids in the PD and PD microdomains design in relation to the modulation of the pore dimensions and the selectivity of the transported molecules, in particular, the siRNAs and miRNAs movement at short- and long-distances. Mutants that express reduced amounts of sphingolipids or that may affect the curved entrance of the tunnel as those described in the former sections would be very interesting to test. These mutants can be also used to investigate the specific lipid environment that sphingolipids provide and that may constitute an important factor determining the correct allocation of transmembrane proteins involved in structural or signaling tasks in the PD domains. This experimentation may help to understand not only the dynamics of miRNAs in systemic defense responses but the sphingolipid contribution to this aspects of plant immunity.

Besides the structural role of sphingolipids to form the PD membranes and microdomains, they can participate in signaling events. In particular, LCB, precursors of complex sphingolipids, can act as second messengers in transduction pathways. LCB are synthesized in the ER by the condensation of serine and palmitoyl-CoA, reaction that is catalyzed by the serine palmitoyltransferase (SPT), yielding keto-sphinganine, which is then reduced to form sphinganine, the simplest LCB that can be enzymatically modified with hydroxylation, phosphorylation and/or unsaturation to form a variety of sphingoid species (Chen et al., 2009). Recent evidences revealed the role of LCB as signaling molecules which are second messengers in the pathway to the programmed cell death that takes place during pathogen infection, the so called Hypersensitive Response (HR) (Peer et al., 2010; Saucedo-García et al., 2011a). In this response, programmed death is manifested only in the cells surrounding the access site of the biotroph pathogens, which lack the capacity of using cell debris as source of nutrients, leaving arrested its dissemination to more distant cells.

### The ER from PD as a local LCB source for propagation to adjacent cells

The fact that the ER is the site of LCB synthesis raises the possibility that in the PD, the local ER is involved in the synthesis of these second messengers with the advantage that in this strategic position, they can reach downstream targets at both contiguous cells, propagating the effect in an efficient way. This could be especially useful in the case of the dissemination of the message to program cell death, since in this case, the establishment of the HR involves the destruction of a limited and therefore controlled number of cells surrounding the access site of the pathogen. In this way, PD aperture among cells close to the pathogen ingress site would be favored by receiving the message eliciting their death to restrain the pathogen spread. In addition, it should be proposed that PD from cells located at longer distances from the pathogen entry site, and which are unexpected to be programmed for death, should maintain the PD in the closed state in order to prevent the transit of the LCB and other signaling molecules. To test this, the mutants *fbr11-1, lcb2a, loh2*, and *acd5*, defective in the response to LCB accumulation or pathogen infection are useful to elucidate the role of LCBs that come from the ER-PD. These studies will contribute to understand the role of PD in the limits for cell death or survival during the HR.

### Involvement of the LCB pathway in the control of PD opening

As second messengers of a transduction route leading to programmed cell death, it is possible that LCB or other pathway components have a direct effect on the proteins that regulate the changes in PD aperture. For example, these sphingolipid precursors activate the salicylic acid response (De la Torre-Hernández et al., 2010; Rivas-San Vicente et al., 2013), a central pathway for local and systemic defense systems. In addition, the C-terminus of Plasmodesmata-Located-Protein 5 (PDLP5), which is rich in cysteine residues, might function as a redox-sensor of Reactive Oxygen Species (ROS) (Wang et al., 2013). These reactive molecules have also been linked to the LCB pathway (Shi et al., 2007; Lachaud et al., 2011; Saucedo-García et al., 2011b). It can be proposed that LCB might be involved in the signaling pathway that leads to the closure of the PD mediated by PDLP5 to avoid the dissemination of bacterial effectors. This could be tested using mutants like *Atlcb2b hp/Atlcb2a, tsc10a, lcb2a-1*, and *sbh1-1, sbh1-2*, shedding light on a possible relation between signaling mediated by sphingolipids and the expression of the gene encoding PDLP5 under pathogen attack. In particular, the study of pathogen proliferation and detection of ROS using these mutants that accumulate less LCB could give information about the operation of the signaling pathway at the PD at early times of pathogen infection.

In addition to the use of mutants defective in genes coding enzymes of sphingolipid metabolism, the pharmacological approach, using inhibitors of the sphingolipid synthesis—fumonisin B1 and myriocin—or degradation, (*N,N*-dimethylsphingosine), constitute an alternative strategy to promote or arrest the accumulation of sphingoid species (Merrill et al., 1993; Shi et al., 2007; De la Torre-Hernández et al., 2010; Saucedo-García et al., 2011a). This alternative has proved to be successful to explore and substantiate the role of LCB as signaling molecules and can be very useful in structural and transduction studies linking sphingolipids to the function of PD and their membrane domains.

## Conclusion

Given the experimental difficulties to dissect the structural organization and function of PD is necessary to approach these studies with diverse strategies. A significant number of *Arabidopsis* mutants defective in genes of sphingolipid metabolism can be used in order to know how these lipids are involved in the assembly and function of PD membranes and their domains.

### Conflict of interest statement

The authors declare that the research was conducted in the absence of any commercial or financial relationships that could be construed as a potential conflict of interest.
